# Discovery of Diverse Natural Products as Inhibitors of SARS-CoV-2 M^pro^ Protease through Virtual Screening

**DOI:** 10.1021/acs.jcim.1c00951

**Published:** 2021-11-22

**Authors:** Jaime Rubio-Martínez, Ana Jiménez-Alesanco, Laura Ceballos-Laita, David Ortega-Alarcón, Sonia Vega, Cristina Calvo, Cristina Benítez, Olga Abian, Adrián Velázquez-Campoy, Timothy M. Thomson, José Manuel Granadino-Roldán, Patricia Gómez-Gutiérrez, Juan J. Pérez

**Affiliations:** † Department of Materials Science and Physical Chemistry, University of Barcelona and the Institut de Recerca en Quimica Teorica i Computacional (IQTCUB), 08028 Barcelona, Spain; ‡ Institute for Biocomputation and Physics of Complex Systems (BIFI), Joint Units IQFR-CSIC-BIFI, and GBsC-CSIC-BIFI, Universidad de Zaragoza, 50018 Zaragoza, Spain; § Departamento de Bioquímica y Biología Molecular y Celular, Universidad de Zaragoza, 50018 Zaragoza, Spain; ∥ Instituto de Investigación Sanitaria de Aragón (IIS Aragon), 50009 Zaragoza, Spain; ⊥ Centro de Investigación Biomédica en Red en el Área Temática de Enfermedades Hepáticas Digestivas (CIBERehd), 28029 Madrid, Spain; # Instituto Aragonés de Ciencias de la Salud (IACS), 50018 Zaragoza, Spain; ∇ Institute of Molecular Biology of Barcelona (IBMB-CSIC), 08028 Barcelona, Spain; ○ Fundación ARAID, Gobierno de Aragón, 50018 Zaragoza, Spain; ◆ Universidad Peruana Cayetano Heredia, San Martín de Porres 15102, Perú; ¶ Departamento de Química Física y Analítica, Facultad de Ciencias Experimentales, Universidad de Jaén, Campus “Las Lagunillas” s/n, 23071, Jaén, Spain; & Department of Chemical Engineering, Universitat Politecnica de Catalunya- Barcelona Tech, Av. Diagonal, 647, 08028 Barcelona, Spain

## Abstract

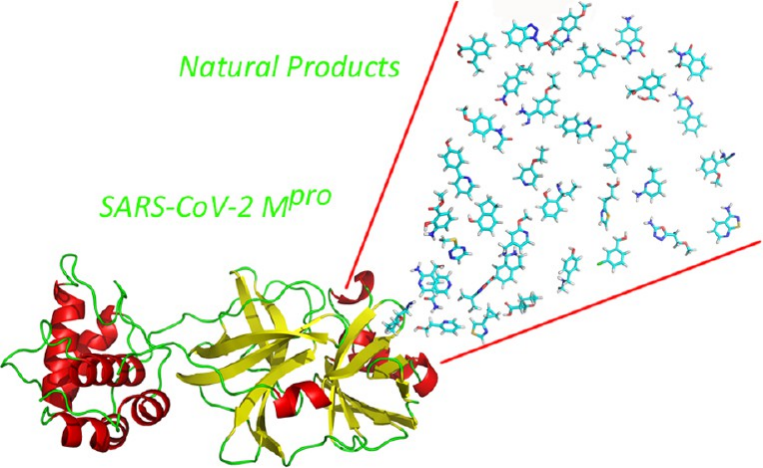

SARS-CoV-2 is a type of coronavirus responsible for the international outbreak of respiratory illness termed COVID-19 that forced the World Health Organization to declare a pandemic infectious disease situation of international concern at the beginning of 2020. The need for a swift response against COVID-19 prompted to consider different sources to identify bioactive compounds that can be used as therapeutic agents, including available drugs and natural products. Accordingly, this work reports the results of a virtual screening process aimed at identifying antiviral natural product inhibitors of the SARS-CoV-2 M^pro^ viral protease. For this purpose, ca. 2000 compounds of the Selleck database of Natural Compounds were the subject of an ensemble docking process targeting the M^pro^ protease. Molecules that showed binding to most of the protein conformations were retained for a further step that involved the computation of the binding free energy of the ligand-M^pro^ complex along a molecular dynamics trajectory. The compounds that showed a smooth binding free energy behavior were selected for in vitro testing. From the resulting set of compounds, five compounds exhibited an antiviral profile, and they are disclosed in the present work.

## 1 Introduction

Coronaviruses, like other members of the coronaviridae family, are enveloped, positive single-stranded RNA viruses that infect a wide range of hosts including avian, swine, and humans.^1^ While most members of the family exhibit mild respiratory effects on humans, the 21st century has witnessed the appearance of new members producing severe respiratory diseases in afflicted individuals. SARS-CoV-1 was identified as the pathogen responsible for the outbreak of a severe acute respiratory syndrome (SARS) in the Guangdong Province, China, in 2002, and 10 years later, MERS-CoV was identified in the sputum of a patient who was retrospectively diagnosed with the Middle East respiratory syndrome (MERS) in Jordania. Both pathogens produced an epidemic that spread across several countries because of international travel of infected persons, which ended about a year later since the outbreak after taking strict measures for infection control.^2^ Beginning in December 2019, a novel coronavirus, designated as SARS-CoV-2, was identified as the pathogen causing an international outbreak of respiratory illness termed COVID-19, which originated in Wuhan, Hubei Province, China. Data gathered on the epidemic suggest that although SARS-CoV-2 exhibits a ∼2% fatality rate, lower than its two ancestors, it is more contagious, resulting in higher overall death rates. This fact forced the World Health Organization to declare SARS-CoV-2 as a pandemic infectious disease of international concern on March 11, 2020.^3^ As of June 20, 2021, there are 178,491,800 confirmed cases of COVID-19 with 3,866,200 confirmed deaths worldwide.^4^

The need for a swift response against COVID-19 prompted to consider drug repurposing as a valuable strategy to cope with the pandemic in a reasonable period of time.^5^ Today, there are a few hundred ongoing clinical trials aimed at assessing the effect of diverse available drugs at different stages of the disease.^6^ A few drugs are currently available for the treatment of COVID-19 patients.^7−9^ Specifically, remdesivir alone^10^ or combined with the Janus kinase inhibitor baricitinib^11^ is the only antiviral agent against SARS-CoV-2 approved with an emergency use authorization for the treatment of patients with severe symptoms. Other antivirals already marketed, like favipiravir^12^ and EIDD-2801,^13^ show mixed evidence, whereas drugs like lopinavir and ritonavir were shown ineffective for the treatment of COVID-19.^14^ Similarly, antimalarial compounds hydroxychloroquine and chloroquine were also shown ineffective.^14,15^ Presently, clinical treatment of COVID-19 is mainly symptomatic using anti-inflammatory agents like dexamethasone^16^ or cytokine inhibitors, combined with antibiotics to treat secondary infections. Accordingly, there remains an urgent need for the development of specific antiviral therapeutics against SARS-CoV-2.

Among the diverse targets available to design antiviral agents, the main proteinase (M^pro^) constitutes an attractive one because it controls the activities of the coronavirus replication complex. The inhibition of M^pro^ was demonstrated to be effective against SARS-CoV-1 in vitro.^17^ Accordingly, several recent studies focus on the design and discovery of inhibitors of the M^pro^ protease for its use as antiviral agents for the treatment of COVID-19. Thus, as a follow-up of previous work devoted to designing suicide inhibitors of M^pro^ in diverse coronavirus, an α-ketoamide has been recently disclosed as a potent inhibitor of the SARS-CoV-2 protease in vitro.^18^ Other researchers have also reported the design of noncovalent inhibitors with a high inhibitory profile against virus duplication in vitro.^19,20^ In the present study, we specifically focus on the identification of natural products, inhibitors of M^pro^ for its use as antiviral agents for the treatment of COVID-19, through the use of virtual screening. Natural products represent an interesting source of molecules for the discovery of antiviral agents.^21,22^ Presently, there are several natural products under efficacy studies for the treatment of COVID-19.^23^ Specifically, diverse plant terpenoids and lignoids have been demonstrated to be efficacious antivirals against SARS-CoV-1, inhibiting viral replication in vitro, with IC_50_ ∼ 1 μM,^24^ and more recently, a series of flavonoids has also been identified as potent inhibitors of SARS-CoV-2 replication in vitro.^25^

Virtual screening is a reliable procedure for a quick and cost-effective way to discover bioactive compounds from large collections against a specific molecular target.^26,27^ A number of in silico studies have recently published on the identification of natural products as inhibitors of M^pro.^^28−30^ However, these studies explore a small set of compounds and do not consider protein plasticity, limiting their scope.^31^ Moreover, most of these studies report predictions that still need to be contrasted experimentally.^32^

The present work reports the results of a robust in silico procedure involving information concerning protein plasticity. Specifically, the study involves a virtual screening of the Selleck database of Natural Compounds containing ∼2000 compounds against a set of diverse conformations of the SARS-CoV-2 M^pro^ protease, characterized from a molecular dynamics study. Accordingly, we first report the characterization of the dynamical profile of protease in its *apo* form, using conventional (cMD) as well as Gaussian accelerated molecular dynamics (GaMD) simulations, in the form of a set of structure representatives. These structures were subsequently used to carry out ensemble docking. Then, the binding free energy of the most promising candidates was assessed using two different procedures to finally provide a shortlist of prospective candidates. These compounds were purchased and tested for their ability to inhibit the M^pro^ protease in vitro. Accordingly, the present work reports the discovery of five SARS-CoV-2 antivirals, inhibitors of M^pro^, identified from a database of natural products using a virtual screening procedure.

## 2 Methods

### 2.1 Computational Studies

#### 2.1.1 System Preparation

The crystallographic structure of the SARS-CoV-2 M^pro^ protease (PDB access code 6Y84) was the starting structure for the present study. Although the crystallographic structure is dimeric, because the active site is not affected by the other copy of the protein, we only considered a monomer for the present study. Hydrogens were subsequently added to every protein residue at their corresponding protonation state at pH 7.0, and side-chain orientations were established using the Protonate3D method^33^ embedded in the molecular operating environment (MOE).^34^ Next, the protein was placed in a cubic box filled with the 4-point, rigid “optimal” point charge (OPC) water molecules,^35^ setting a minimum distance of 15 Å between the solute and the box walls. Water molecules closer than 1.2 Å to any complex atom were removed. Then, two Na^+^ ions were added to neutralize the system, at the positions of the lowest electrostatic potential using the Leap module of AMBER18.^36^ All calculations were done using the ff19SB force field^37^ with a cutoff of 10 Å for noncovalent interactions and using the particle mesh Ewald (PME) method^38^ for the treatment of electrostatic interactions.

#### 2.1.2 Energy Minimization

Before starting the molecular dynamics calculations, the structure was first relaxed to eliminate possible steric clashes in a multistep minimization procedure of 5000 steps each using the steepest descent method. First, only water molecules and ions were allowed to relax by keeping all the atoms of the protein fixed, applying a harmonic positional restriction of 5 kcal/mol·Å^–2^. In the second step, only the main atoms of the protein were kept fixed with the same harmonic positional restrain as before. Finally, in the third step, all the atoms were allowed to move.

#### 2.1.3 Molecular Dynamics Simulations

After minimization, the system was heated to 300 K stepwise at a rate of 30 K every 20 ps, fixing the main atoms of the protein with a harmonic positional restriction of 0.5 kcal/mol·Å^–2^ using the Langevin thermostat algorithm with a collision frequency of 2 ps^–1^ under the NVT ensemble (from now on, *heating*). Subsequently, 2 ns simulation was performed at constant pressure (NPT ensemble), keeping fixed the main atoms of the protein with a harmonic positional restriction of 0.1 kcal/mol·Å^–2^ for density equilibration (from now on, *density equilibration*). Finally, conventional molecular dynamics (cMD) and GaMD of 500 ns length were carried out within the NVT ensemble in duplicate to increase the explored conformational space of the system.^39^ In the case of GaMD simulations, after density equilibration, an intermediate step of 20 ns was performed to obtain the initial statistical analysis of the dual boost potential. The upper limit of the standard deviation of the total potential boost (σ_0P_) was set to 3, and the upper limit of the standard deviation of the dihedral potential boost (σ_0V_) was set to 5. In these simulations, a cutoff of 11 Å was used together with a switch function at 8 Å.

#### 2.1.4 Root-Mean-Square Deviation and Root-Mean-Square Fluctuation)

Root-mean-square deviation (RMSD) along the simulation time was computed using the cpptraj module^40^ from AMBER18 for all the molecular dynamics trajectories to assess the structural stability of the systems along time. RMSD was computed using the last minimized structure as a reference. However, an iterative procedure was used to select the alpha carbon atoms (Cα) with the smallest fluctuations. Thus, in the first step, all Cα of the diverse residues was used to reorient the structures. The resultant superposed trajectories were used to calculate the root-mean-square fluctuation (RMSF) for each of the residues of the protein using cpptraj. Residues with an RMSF smaller than the first threshold were selected to be used in the next calculation of the RMSD and so on. Thus, for the first step, all the Cα atoms were used in the superposition, but in the next three steps, a cutoff of 2.0, 1.0, and 0.5 Å, respectively, on the RMSF values were used to select the Cα to be superposed (Figures S1 and S2 of the Supporting Information). In the last step, a total of 35 amino acids met the desired criteria. This iterative process provides a set of amino acids with small fluctuations along the full MD that can be used to obtain information of the local conformational flexibility for the nonsuperposed residues.

#### 2.1.5 Cluster Analysis

In order to select a group of structures representing the greatest structural diversity of the binding site of the M^pro^ protease, similar structures in both the cMD and GaMD simulations were grouped into 15 different clusters using the average linkage algorithm,^41^ as implemented in the cpptraj module of AMBER18.^36^ For this process, the RMSD of the Cα located in the binding site with a larger RMSF was used as the distance. A total of 54 amino acids were selected (Figure S2 of the Supporting Information).

#### 2.1.6 Principal Component Analysis

In order to determine and analyze the extent of the conformational space accessed in different approaches and understand how different the representatives selected by our clustering methodology are, we used the principal component analysis (PCA). This statistical technique is routinely applied to reduce the number of dimensions needed to describe protein motions from the largest to the smallest spatial scales. First, a covariance matrix was constructed including all the structures obtained in the different molecular dynamics and using the atomic coordinates of the Cα atoms of the same residues as in the clustering process. Subsequently, the covariance matrix was diagonalized to produce a set of eigenvectors or principal components (PC^(*i*)^, *i* = 1, *N*), with *N* being the number of selected residues of the protein (in our case, 54 residues), as well as their corresponding eigenvalues, λ^(*i*)^. After the eigenvalues are rank-ordered, the first components define the “essential” space or motions of the protein.^42^

#### 2.1.7 Virtual Screening

**Figure 1:**
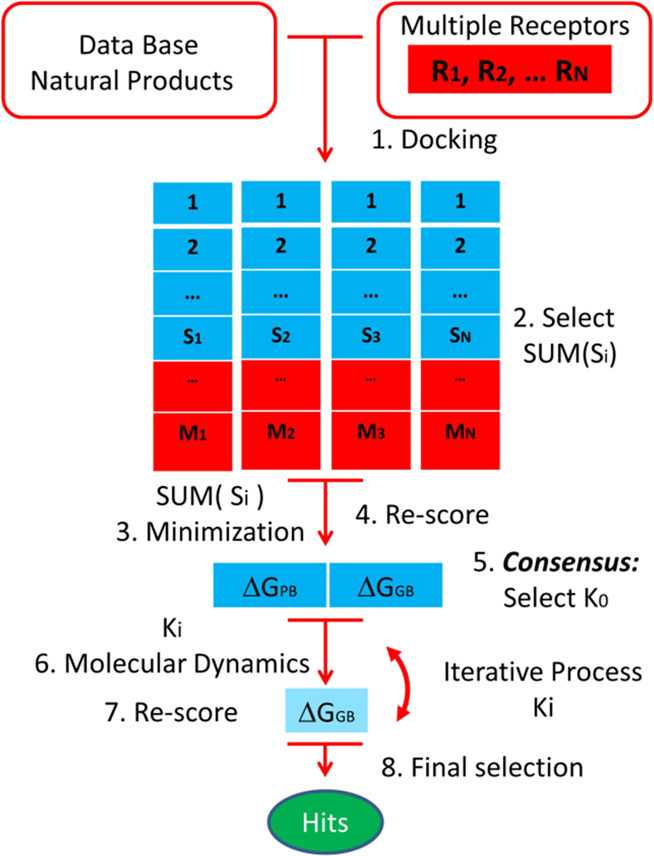
Multistep virtual screening flowchart.

A multistep virtual screening procedure was performed on each of the seven M^pro^ representatives selected, which is summarized in Figure 1. In step 1, the QVina2 software^43^ was used to dock 1872 molecules of the natural products database from Selleck Chemicals^44^ in each of the seven target representatives. Molecules from the database had been previously processed to have the right protonation state and their geometries optimized using the MOE software.^34^ The docking process was carried out using a rectangular box of dimensions 32.25 × 31.5 × 35.25 Å, centered in the middle of the plane defined by the Cα of residues Cys^145^, Leu^27^, and His^41^. In step 2, we selected these complexes with a scoring function higher than −7.0 kcal/mole in each M^pro^ representative. In step 3, the Antechamber and Leap modules of the Amber18 package^36^ were used to parameterize the ligands with gaff2 force field,^45^ solvate the complexes in a box of TIP3P water molecules,^46^ and add counterions to the complexes. The ff14SB force field^47^ was used to parameterize the protein. Then, each complex was relaxed in a three-step minimization process using 5000 steps in each by means of the steepest descent method. First, only the water molecules and ions were allowed to relax by keeping all the atoms of the protein and ligand fixed, applying a harmonic positional restriction of 5 kcal/mol·Å^–2^. In the second step, only the main atoms of the protein were kept fixed, with a harmonic positional restrain of 5 kcal/mol·Å^–2^, allowing the ligand to move freely. Finally, in the third step, all the atoms were allowed to move. In the fourth step of the process, the free energy of binding Δ*G*_binding_ (GB) was computed for all the minimized structures using both the molecular mechanics Poisson–Boltzmann surface area (MMPBSA)^48^ and the molecular mechanics generalized Born surface area (MMGBSA)^49^ procedures. These calculations provide a new scoring to rank order the ligands. Next, we introduced in step 5 a *consensus* criterion to select those complexes that will be studied further using molecular dynamics simulations in step 6. Then, in step 7, a new rank-ordered list is obtained after applying the MMGBSA approach to the full-length molecular dynamics simulation. Next, an iterative process was done, where, at each step, for the best compounds obtained in the previous step, their molecular dynamics length was extended and the GB recalculated. In the last step, a final selection of compounds is performed based on their GB for the more extended molecular dynamics and the analysis of the ligand–receptor interactions at the binding site.

#### 2.1.8 Binding Free Energy Computation

The binding free energy was computed using the MMPBSA and the MMGBSA procedures,^50^ as implemented in the AMBER18 package.^36^ In both methods, the free binding energy is computed according to the following equation:
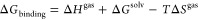
where Δ*H*^gas^ is the gas-phase interaction energy calculated by summing the internal energy, noncovalent van der Waals (Δ*H*_vdW_^gas^) energy, and electrostatic (Δ*H*_elec_^gas^) molecular mechanics energy. On the other hand, Δ*G*^solv^ is computed as the sum of polar (Δ*G*_polar_^solv^) and nonpolar terms (Δ*G*_nonpolar_^solv^). The former term is calculated numerically by solving the Poisson–Boltzmann (PB) equation^51^ or its simplified form and the generalized Born (GB) method^52^ for both the MMPBSA and MMGBSA algorithms, respectively. In the present work, we used the Onufriev–Bashford–Case (OBC) GB method (igb = 2).^53^ Regarding Δ*G*_nonpolar_^solv^, it is calculated using the following equation:
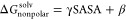
where SASA is the solvent-accessible surface area calculated using the Linear Combinations of Pairwise Overlaps (LCPO) method,^54^ and the values for γ and β constants were set to 0.00542 kcal/mol·Å^2^ and 0.92 kcal/mol for MMPBSA^48^ and 0.0072 kcal/mol·Å^2^ and 0 kcal/mol for MMGBSA,^49^ respectively. All the calculations were carried out with the MMPBSA.py program.^55^

### 2.2 Experimental Procedure

#### 2.2.1 SARS-CoV-2 M^pro^ Expression and Purification

M^pro^ was expressed in a pET22b plasmid transformed into BL21 (DE3) gold *Escherichia coli* strain. Small-scale cultures grown in LB/ampicillin (100 μg/mL) at 37 °C overnight were employed for inoculating 4 L large-scale cultures of LB/ampicillin (100 μg/mL) incubated at 37 °C until reaching an OD close to 0.6 at 600 nm. The protein expression was induced with 1 mM isopropyl 1-thio-β-D-galactopyranoside (IPTG) at 18 °C for 5 h. Cells were harvested by centrifugation at 4 °C for 10 min at 10,000 rpm (Beckman Coulter Avanti J-26 XP Centrifuge) and resuspended in lysis buffer (sodium phosphate 50 mM, pH 7, sodium chloride 500 mM). Cells were lysed by sonication (Sonics Vibra-Cell Ultrasonic Liquid Processor) on ice, adding benzonase 20 U/mL (Merck-Millipore) and lysozyme 0.5 mg/mL (Carbosynth). Cell debris was removed by centrifugation at 4 °C for 30 min at 20,000 rpm and by subsequent filtration (0.45 μm pore membrane). Affinity chromatography (ÄKTA FPLC System, GE Healthcare Life Sciences) using a cobalt HiTrap TALON column (GE Healthcare Life Sciences) allowed fast purification in a single chromatographic step, applying an imidazole 10–250 mM gradient. Purity was assessed by sodium dodecyl sulfur polyacrylamide gel electrophoresis (SDS-PAGE), and pure protein fractions were pooled and dialyzed to remove imidazole in buffer (sodium phosphate 50 mM, pH 7, sodium chloride 150 mM). The protein concentration was quantitated using an extinction coefficient of 32,890 M^–1^ cm^–1^ at 280 nm. Protein identity was assessed by mass spectrometry (LC-ESI-MS/MS).

#### 2.2.2 SARS-CoV-2 M^pro^ Proteolytic Activity Assay

A continuous assay based on Förster resonance energy transfer (FRET) to measure the catalytic activity of M^pro^ in vitro was implemented using the substrate (Dabcyl)KTSAVLQSGFRKME(Edans)-NH2 (Biosyntan GmbH). The enzymatic reaction was initiated by adding the substrate at 20 μM (final concentration) to the enzyme at 0.2 μM (final concentration) in a final volume of 100 μL. The reaction buffer was sodium phosphate 50 mM, pH 7, NaCl 150 mM. For compounds dissolved in pure dimethyl sulfoxide (DMSO) as a stock solution, a constant DMSO percentage (2.5%) was maintained in all assays. Fluorescence emission was measured in a FluoDia T70 microplate reader (Photon Technology International) for 20 min (excitation wavelength, 380 nm; emission wavelength, 500 nm). The initial slope of the time evolution curve of the fluorescence emission signal provided a direct quantification of the enzymatic activity. The Michaelis–Menten constant, *K*_m_, and the catalytic rate constant or turnover number, *k*_cat_, were previously estimated (*K*_m_ = 11 μM and *k*_cat_ = 0.040 s^–1^).

#### 2.2.3 SARS-CoV-2 M^pro^ Inhibition Assay

The in vitro inhibition potency of the compounds against M^pro^ was assessed through the estimation of the inhibition constant, *K*_i_, and the half-maximal inhibitory concentration, IC_50_, from experimental inhibition curves. Inhibition curves were obtained by measuring the enzyme activity (at fixed 0.2 μM enzyme concentration and fixed 20 μM substrate concentration) as a function of the compound concentration (serial twofold dilution from 125 to 0 μM), maintaining the percentage of DMSO constant (2.5%) for compounds dissolved in DMSO. The enzymatic activity was quantitated as the initial slope of the substrate fluorescence emission time evolution curve and was plotted as a function of the compound concentration. The ratio between the activity (slope) in the presence and absence of compounds provides the residual percentage of activity at a given compound concentration. Nonlinear regression analysis employing a simple inhibition model (considering inhibitor depletion because of enzyme binding) allowed us to estimate the apparent inhibition constant, *K*_i_^app^, for each compound, according to eq 1:
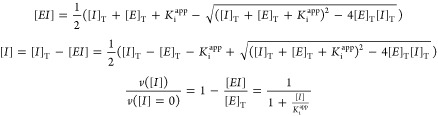
1where [*EI*] is the concentration of the enzyme-inhibitor complex, [*E*]_T_ and [*I*]_T_ are the total concentrations of enzyme and inhibitor, respectively, *K*_i_^app^ is the apparent inhibition constant for the inhibitor, [*I*] is the concentration of the free inhibitor, and *v* is the initial slope of the enzymatic activity trace at a given (free) inhibitor concentration [*I*] (or the total inhibitor concentration [*I*]_T_). No approximation for the free inhibitor concentration (e.g., assuming to be equal to the total inhibitor concentration) was made, thus having general validity for any total enzyme and inhibitor concentration and any value of the inhibition constant. In addition, if the inhibitor acts through a purely competitive mechanism, the previous equation can be substituted by eq 2:
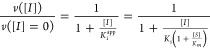
2

where *K*_i_ is the intrinsic (i.e., substrate concentration-independent) inhibition constant, *K*_m_ is the Michaelis–Menten constant for the enzyme-substrate interaction, and [*S*] is the substrate concentration. By approximating the free compound concentration by the total compound concentration and neglecting ligand depletion, the *K*_i_^app^ in eq 2 is equivalent to the IC_50_. It should be noted that as the IC_50_ is an assay-dependent inhibition potency index (among other parameters, it depends on the enzyme and substrate concentrations, as well as on the *K*_m_), the intrinsic inhibition constant is a better inhibition potency index.

#### 2.2.4 Purity of the Compounds Tested

The 11 compounds tested in the present study were purchased from Selleck Chemicals (Houston, TX, USA). All compounds are >95% pure by high-performance liquid chromatography (HPLC). HPLC traces for representative compounds are included in the Supporting Information.

## 3 Results and Discussion

### 3.1 Selection of Structures Representing M^pro^ Plasticity

**Figure 2:**
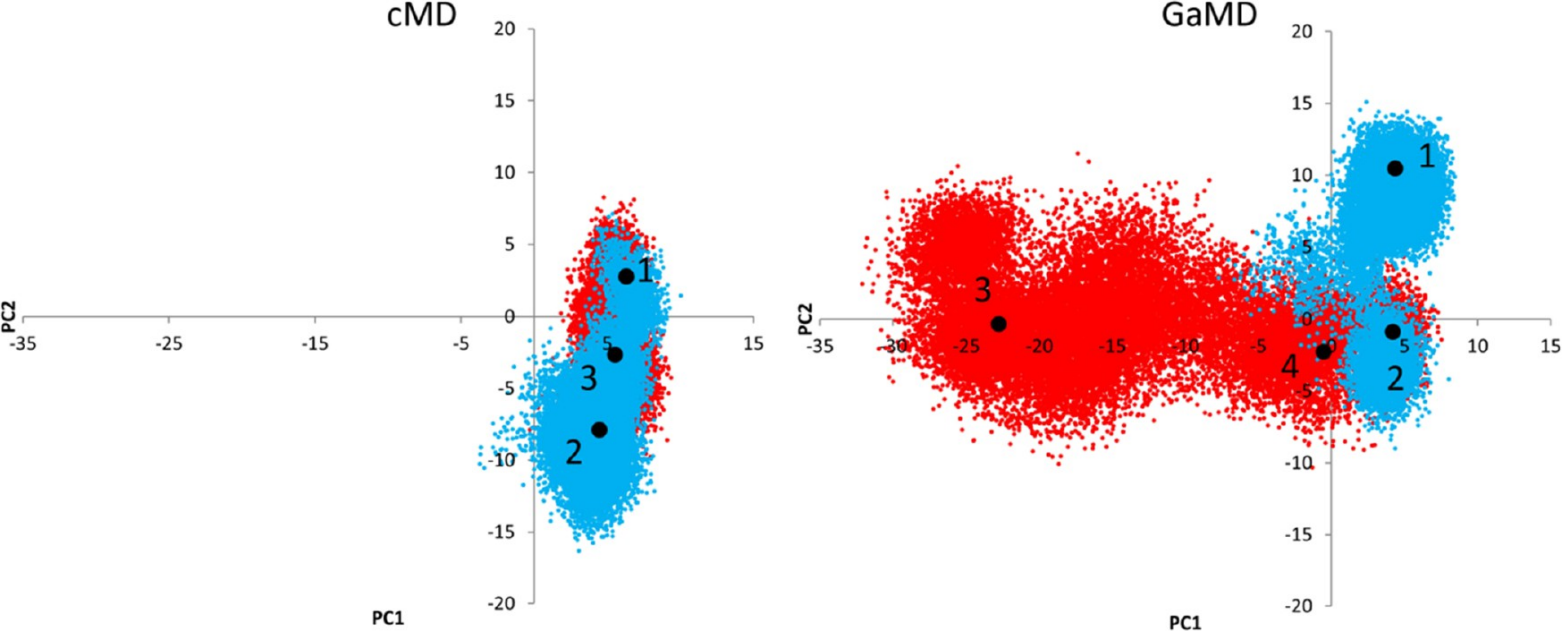
Representation of the first two PCs sampled using cMD and GaMD approaches. Blue and red indicate the two different molecular dynamics simulations. The big black points are the positions of the selected representatives for the clusters with more than 10% of the total population; three for the cMD and four for the GaMD.

A clustering process was performed for both cMD and GaMD calculations separately, as explained in the Section 2 to identify representative structures of the most populated clusters. We previously had performed an iterative process to select the set of atoms to be involved in the superposition process, bearing in mind to cover the maximum conformational diversity of the binding site in the selected representatives (Figure S1 of the Supporting Information). Thus, we iteratively selected the atoms involved in the superposition process according to their RMSF (Figure S2 of the Supporting Information). After the last step, 35 amino acids located in the binding site with small fluctuations along the MD trajectory were selected to superimpose the structures (Figure S3 of the Supporting Information). Once the superposition was performed using the corresponding Cα, the RMSD of a total of 54 amino acids located in the binding site with large RMSF values was used as the distance for the clustering process (Figures S2 and S3 of the Supporting Information). Three and four representatives were selected for both the cMD and GaMD, respectively, representing clusters with more than 10% population. Although the assessment of the conformational diversity of our selected structures can be done by visual inspection (Figure S4 of the Supporting Information), we used PCA to obtain a clearer picture. For this purpose, we analyzed the RMSF of the amino acids located on the binding site. The amino acids with lower RMSF values were used for the superimposition of the structures, whereas those with larger RMSF values were used for the computation of the covariance matrix (Figure S3 of the Supporting Information). As shown in Figure 2, the conformational space covered by cMD and GaMD is markedly different, a fact that is further stressed after using two MD runs for each approach. Thus, the representatives selected will describe a broad range of situations where the ligands can bind.

### 3.2 Virtual Screening Targeting the SARS-Cov-2 M^pro^ Protease

The QVina2 software^43^ was used to perform ensemble docking of the diverse molecules from the Natural Product database onto the seven M^pro^ structural representatives and compute their corresponding scoring function values. For each structure representative, those ligand-M^pro^ complexes with a scoring function lower or equal to an established threshold were rank-ordered and conserved for further analysis. A threshold of −7.0 kcal/mol was established after the analysis of the results produced for the most populated cluster representative identified from the cMD. Specifically, the plot of the cumulative number of complexes obtained versus their scoring function value (see in Figure S5 of the Supporting Information) shows that there are already around 500 complex values, with −7.0 kcal/mol or lower, that represents a number large enough to include chemical diversity and permits to keep the computational cost reasonable. Complexes selected may include more than one pose per compound, and actually, the same compound may appear in the rank-ordered list of different representatives. The application of the threshold to the different M^pro^ structures yields different numbers of complexes for each structure. Specifically, 513, 878, and 637 for the three cMD representatives and 558, 1840, 949, and 293 for the four GaMD representatives.

Ligand–receptor complexes selected from the docking process were subsequently subjected to a minimization process in explicit water, allowing complete conformational freedom for both the ligand and the protease. The binding energy of the minimized structures was subsequently computed using the end-point methodologies MMPBSA^48^ [Δ*G*_binding_(PB)] and MMGBSA^49^ [Δ*G*_binding_ (GB)]. Thus, at the end of this process, we produced two rank-ordered lists for each M^pro^ representative structure, giving a total of 14 lists. The selection of the set of prospective binders was performed, following a *consensus* approach. The procedure is based on the assumption that the larger the number of target conformations a ligand binds, the higher are its chances of being a hit. Accordingly, we did not select directly compounds with the lowest binding energy, but those ligands that exhibit binding to diverse conformations of the target within the threshold. Using this criterion, we selected 47 compounds that exhibit binding to all 7 structural representatives of the target, together with additional 21 compounds that exhibit binding to six out of seven structural representatives, producing a total of 68 compounds. For each compound, we selected the complex structure with the lowest binding energy for further studies.

The 68 selected complex structures were prepared for the production step, as described in Section 2.1.3. Thus, a *heating* from 0 K to 300 K and a *density equilibration* for each one were carried out before a 100 ns of production molecular dynamics simulation. After completion, the Δ*G*_binding_ (GB) time evolution of every compound was computed using the MMGBSA approach. The analysis of these plots shows that 38 out of the 68 ligand–protease complexes exhibit a smooth fluctuating behavior during the last 20 ns. In order to reduce the final number of candidates, these 38 complexes were selected to extend their MD simulations up to 200 ns. After the analysis of the Δ*G*_binding_ (GB) behavior and using the same criterion, 21 ligand–protease complexes were selected for another round of MD simulations, extending them up to 500 ns. In the final step, using the same criterion, only 11 complexes were selected for extending their MD simulations up to 1.5 μs to check the smooth behavior of the free energy of binding previously observed.

**Figure 3:**
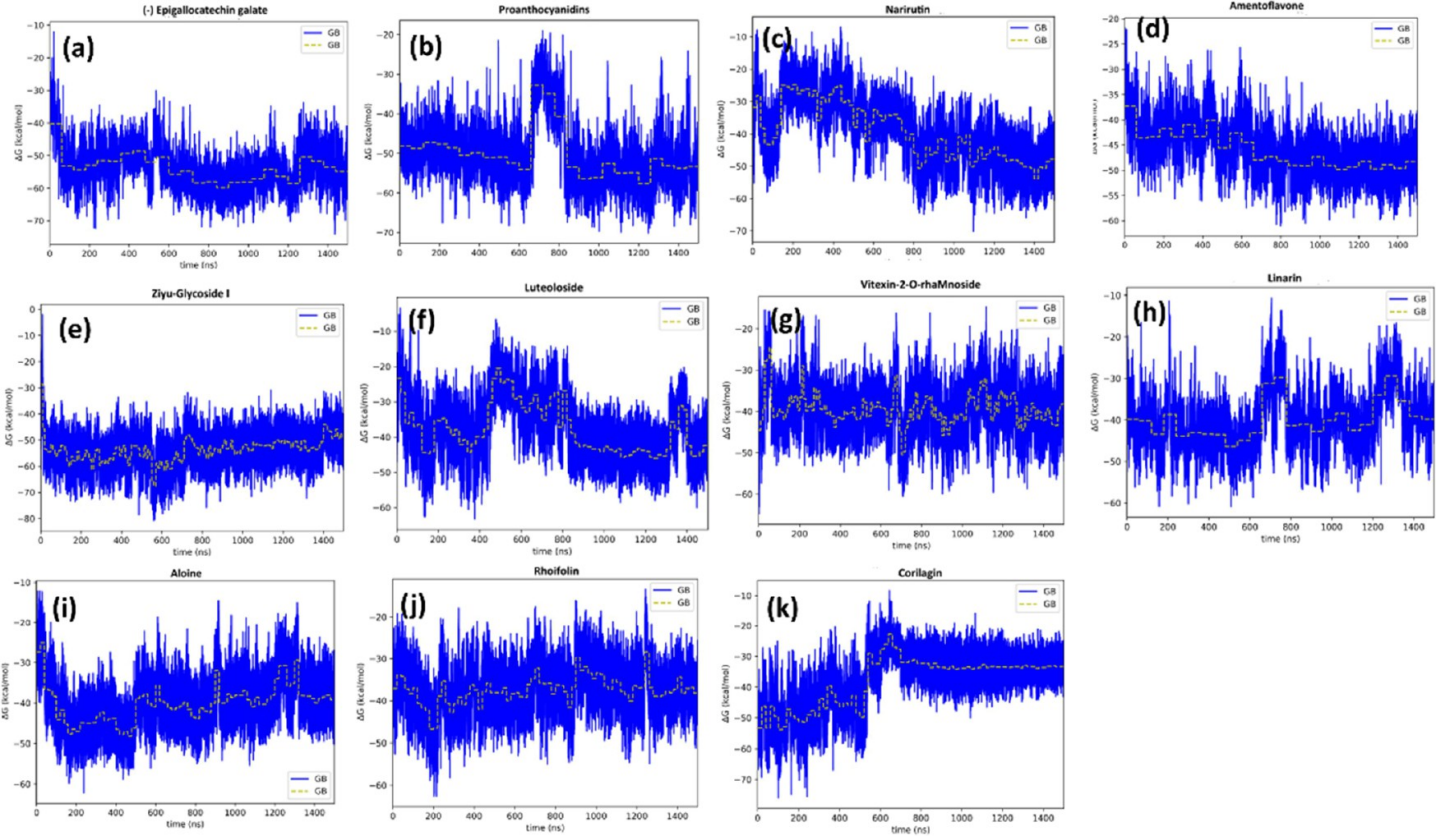
(a–k) Time evolution of the binding free energy of the 11 compounds selected from the virtual screening process.

Compounds were selected after the inspection of the time evolution of the binding free energy during the MD simulation. A smooth behavior with small fluctuations around the mean is considered as the indication of good candidates, although some of the compounds show important fluctuations that are corrected at the end of the respective simulations. The time evolution of Δ*G*_binding_ (GB) for the 11 selected complexes using the MMGBSA approach is shown in Figure 3a–k.

**Figure 4:**
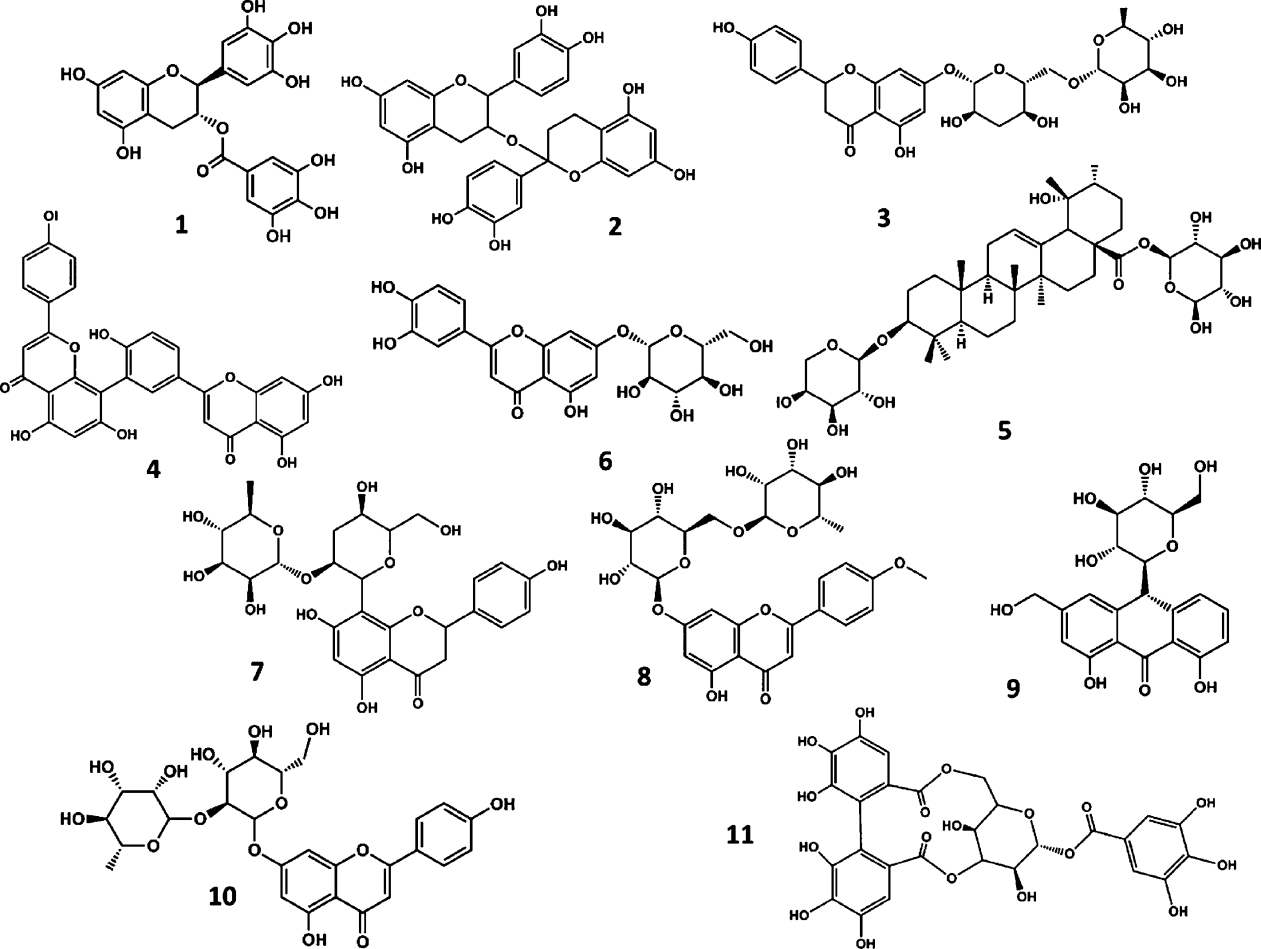
Chemical structures of the natural compounds identified as prospective hits targeting the M^pro^ protease from the virtual screening process. (−) Epigallocatechin gallate (**1**), proanthocyanidins (**2**), narirutin (**3**), amentoflavone (**4**), ziyu-glycoside I (**5**), luteoloside (**6**), vitexin-2-O-rhaMnoside (**7**), linarin (**8**), aloin (**9**), rhoifolin (**10**), and corilagin (**11**)

After the analysis of the time evolution plots, 11 compounds were selected as prospective candidates resulting from the virtual screening process, including (−) epigallocatechin gallate (**1**) (this refers to (2*R*,3*R*)-5,7-dihydroxy-2-(3,4,5-trihydroxyphenyl)-3,4-dihydro-2H-1-benzopyran-3-yl 3,4,5-trihydroxybenzoate, the major polyphenolic catechin found in green tea), proanthocyanidins (**2**), narirutin (**3**), amentoflavone (**4**), ziyu-glycoside I (**5**), luteoloside (**6**), vitexin-2-O-rhaMnoside (**7**), linarin (**8**), aloin (**9**), rhoifolin (**10**), and corilagin (**11**) (Figure 4).

### 3.3 In Vitro M^pro^ Inhibitory Activity of Candidate Compounds

The 11 prospective candidates identified from the virtual screening process were purchased and tested in an in vitro assay. Specifically, the inhibitory potential of the compounds against recombinant SARS-CoV-2 Mpro was tested using the FRET assay, as described in the Section 2. Five compounds showed specific inhibitory activity, with substrate concentration-independent inhibition constants (*K*_i_) ranging from 7.8 μM for (−) epigallocatechin gallate to 82 μM for aloin. The remaining seven compounds did not yield detectable inhibitory activities at concentrations below 125 μM. Table 1 lists their measured activity together with their binding energy computed, as described in the Section 2. Inhibition curves are shown in Figure S6 of the Supporting Information.

**Table 1:** In Vitro Inhibition Values Exhibited by the Diverse Compounds Purchased Rank-Ordered by their Computed Binding Energies^a^

compound	*K*_i_ (μM)	IC_50_ (μM)	Δ*G*_binding_ (GB) (kcal/mol)
(−) epigallocatechin gallate	7.8	22	–54.6
proanthocyanidins	*	*	–52.9
narirutin	*	*	–48.9
amentoflavone	10	28	–48.5
ziyu-glycoside I	*	*	–48.1
luteoloside	*	*	–43.6
vitexin-2-O-rhamnoside	23	65	–40.9
linarin	*	*	–39.6
aloin	34	96	–38.9
rhoifolin	82	230	–36.9
corilagin	*	*	–33.4

a Compounds with no detectable inhibitory activity at concentrations below 125 μM are marked with an asterisk.

Inspection of Table 1 shows that there is a correlation for the active compounds between the computed binding energy to the M^pro^ protease and their inhibitory capacity in vitro. However, despite having reasonable binding affinities, several of the listed compounds do not exhibit inhibitory activity. Actually, the procedure followed to identify active compounds yields a 45% success rate, as previously found in similar studies.^56,57^ This can be attributed to diverse factors related to the physicochemical properties of the compounds like solubility or the lipophilicity profile among others that may prevent reaching the target in the conditions of the experiment. Among the compounds reported in the present study, (−) epigallocatechin gallate^58−60^ and rhoifolin^25^ have already been reported as M^pro^ protease inhibitors from screening studies. Moreover, vitexin has also been proposed as a prospective M^pro^ inhibitor from modeling studies.^61^ The rest of the active compounds are disclosed in the present work for the first time.

As previously shown, the procedure used in the present work to select prospective candidates is based on the behavior of the time evolution of the ligand-M^pro^ complex binding free energy. Fluctuations are associated to the movement the ligands experienced inside the binding pocket. Specifically, when the time evolution of the binding free energy is smooth, it fluctuates ∼20 kcal/mol around an average value, and it is stable with time. These fluctuations can be associated with ligand rattling inside the binding pocket but bound in a specific pose. Ligands of this category are considered for experimental evaluation. Larger fluctuations may be associated with a lack of steric complementarity between the ligand and the protein binding pocket so that ligands have lower chances to become hits. In contrast, abrupt changes are associated with the accommodation of the ligand inside the binding pocket. When the subsequent behavior is stable, ligands are considered for experimental evaluation. In contrast, if fluctuations persist, ligands are discarded as candidates. Finally, behaviors where the binding free energy does not exhibit a stable average behavior have lower chances to become hits. In summary, this procedure relies on the ligand-bound residence time as the indicator of the chances for a ligand to be a hit and represents a more robust discrimination procedure than using only the predicted binding free energy. Thus, (−) epigallocatechin gallate, the most active compound identified in this study, exhibits a smooth time evolution (Figure 3a) with fluctuations around 20 kcal/mol. Similarly, plots of the other active compounds, including amentoflavone (Figure 3d), vitexin-2-rhamnoside (Figure 3g), aloin (Figure 3i), and rhoifolin (Figure 3j) show stable behaviors. The only exception to this criterion is represented by ziyu-glucoside I that despite exhibiting a stable time evolution (Figure 3g), the compound turns out to be nonactive in the experimental test.

Regarding the nonactive compounds, the inspection of the time evolution of the free energy of binding can provide hints of their lack of inhibitory capacity. Specifically, the inspection of the proanthocyanidins plot (Figure 3b) shows a large fluctuation around 700 ns as a sign of instability. Although the average binding free energy comes back to previous values, the system exhibits fluctuations larger than 20 kcal/mole. In this case, the compound turns out to be nonactive, despite exhibiting a good binding free energy. The plot of linarin (Figure 3h) shows several fluctuations that suggest positional changes of the ligand inside the binding pocket that can explain its lack of activity. The behavior of corilagin (Figure 3k) suggests that the ligand, despite showing a stable behavior after 600 ns, is subjected to conformational changes that produce a loss of stability from the starting position. On the other hand, narirutin and luteoloside exhibit a time evolution binding free energy plots (Figures 3c,f, respectively) that are not converged after 1.5 μs simulation time.

**Figure 5:**
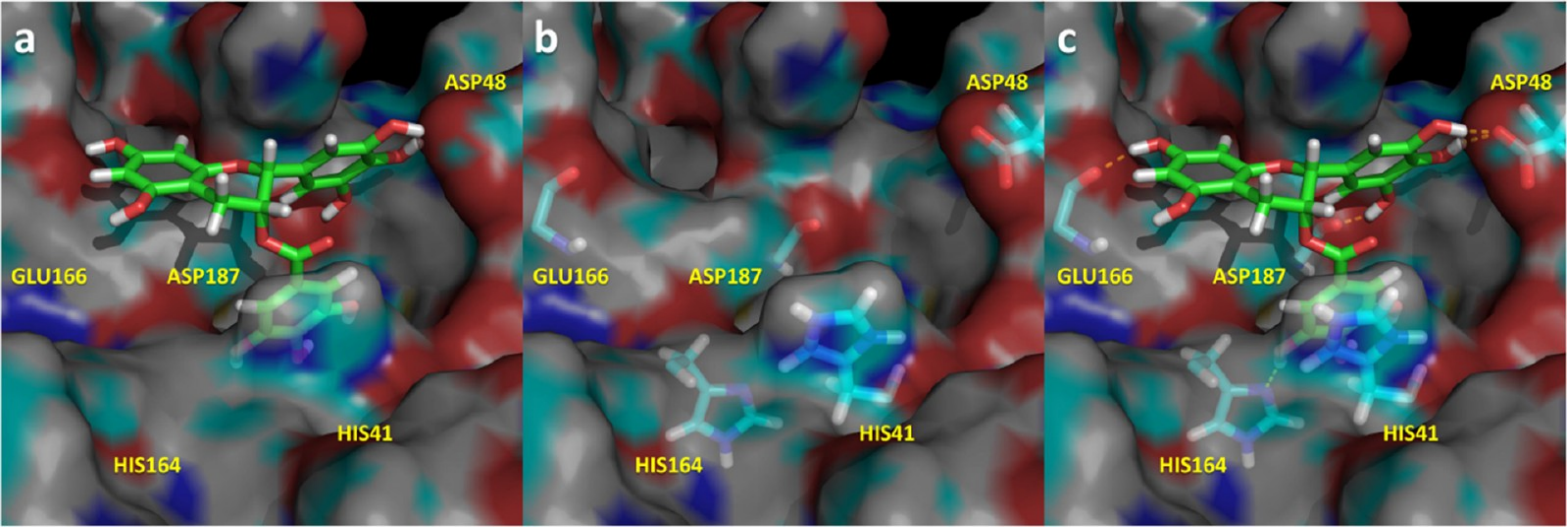
Spatial representation of the complex M^pro^-(−) epigallocatechin gallate in its last snapshot of the 1.5 μs molecular dynamics. (a) Ligand bound to the binding pocket; (b) spatial distribution of the most important residues that interact with the ligand; (c) ligand–protease hydrogen bonds in yellow.

The prospective bound conformation of the five ligands found to be active onto the active site of M^pro^ is shown in Figures 5^6^,7,8,9. Specifically, these structures correspond to the last snapshot of the corresponding 1.5 μs molecular dynamics trajectory. The inspection of Figures 5^6^,7,8,9 suggests that ligands occupy common spots of the binding site, including the S1′, S1, and/or S2 subsites,^20^ although some of the residues involved in ligand–enzyme interactions can be different for the diverse ligands. Thus, all the ligands occupy the S1′ subsite and the location of the catalytic dyad Cys^145^ and His^41^, establishing hydrogen-bond interactions with the former and quadrupole–quadrupole interactions with the latter. Furthermore, other residues like Glu^166^ (located in the S1 subsite) or Gln^142^ together with Asp^187^ (located in the S2 subsite) establish hydrogen bonds with some of the ligands, as summarized in Table S1 of the Supporting Information. Interestingly, the ligand amentoflavone because of its size also occupies the S4 subsite of the binding site. All these residues have already been reported as important for designing novel M^pro^ inhibitors.^20,62^

**Figure 6:**
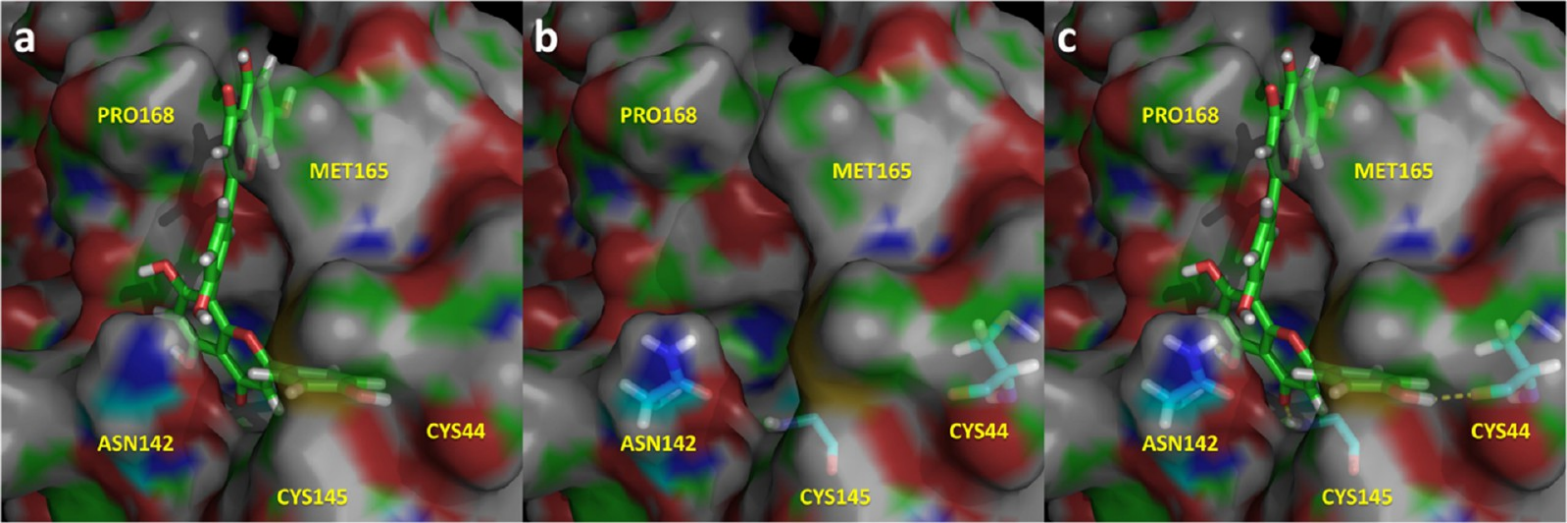
Spatial representation of the complex M^pro^-amentoflavone in its last snapshot of the 1.5 μs molecular dynamics. (a) Ligand bound to the binding pocket; (b) spatial distribution of the most important residues that interact with the ligand; (c) ligand–protease hydrogen bonds in yellow.

**Figure 7:**
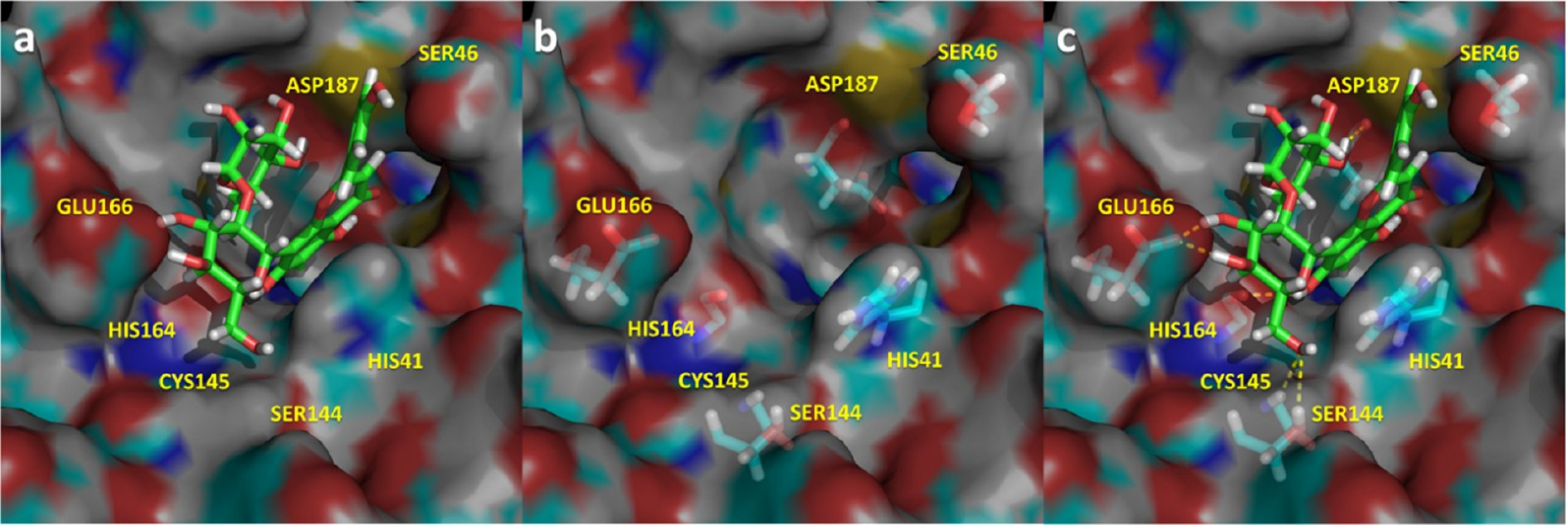
Spatial representation of the complex M^pro^-Vitexin-2-*O*-rhamnoside in its last snapshot of the 1.5 μs molecular dynamics. (a) Ligand bound to the binding pocket; (b) spatial distribution of the most important residues that interact with the ligand; (c) ligand–protease hydrogen bonds in yellow.

**Figure 8:**
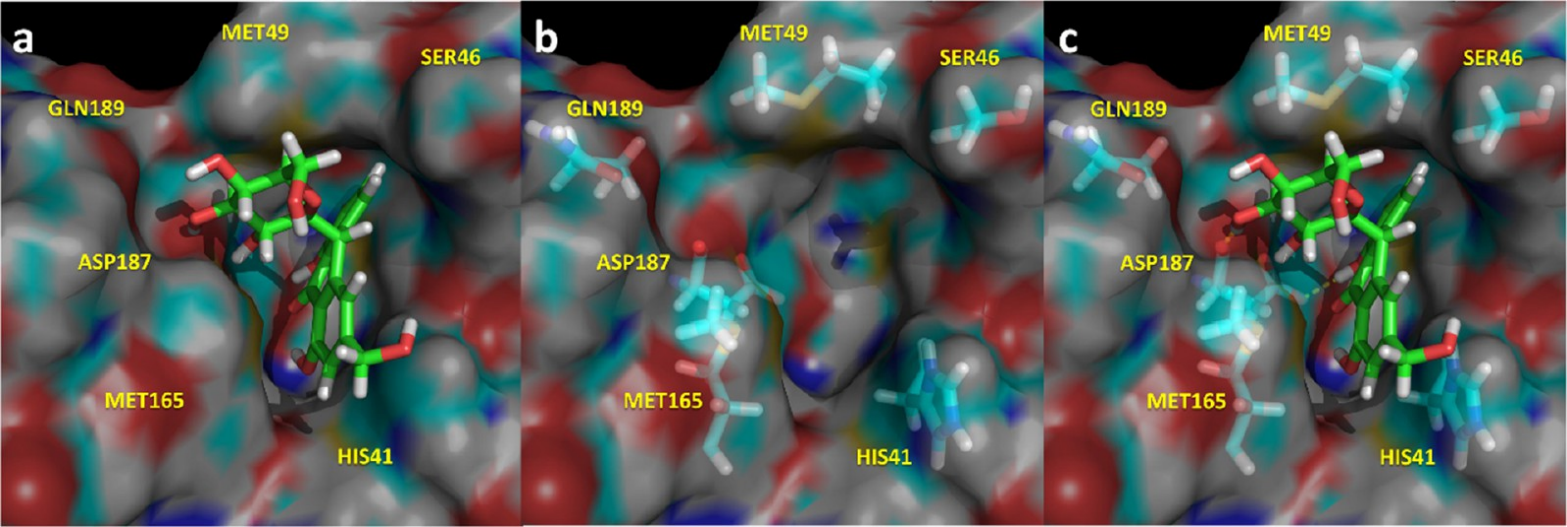
Spatial representation of the complex M^pro^-Aloin in its last snapshot of the 1.5 μs molecular dynamics. (a) Ligand bound to the binding pocket; (b) spatial distribution of the most important residues that interact with the ligand; (c) Ligand–protease hydrogen bonds in yellow.

**Figure 9:**
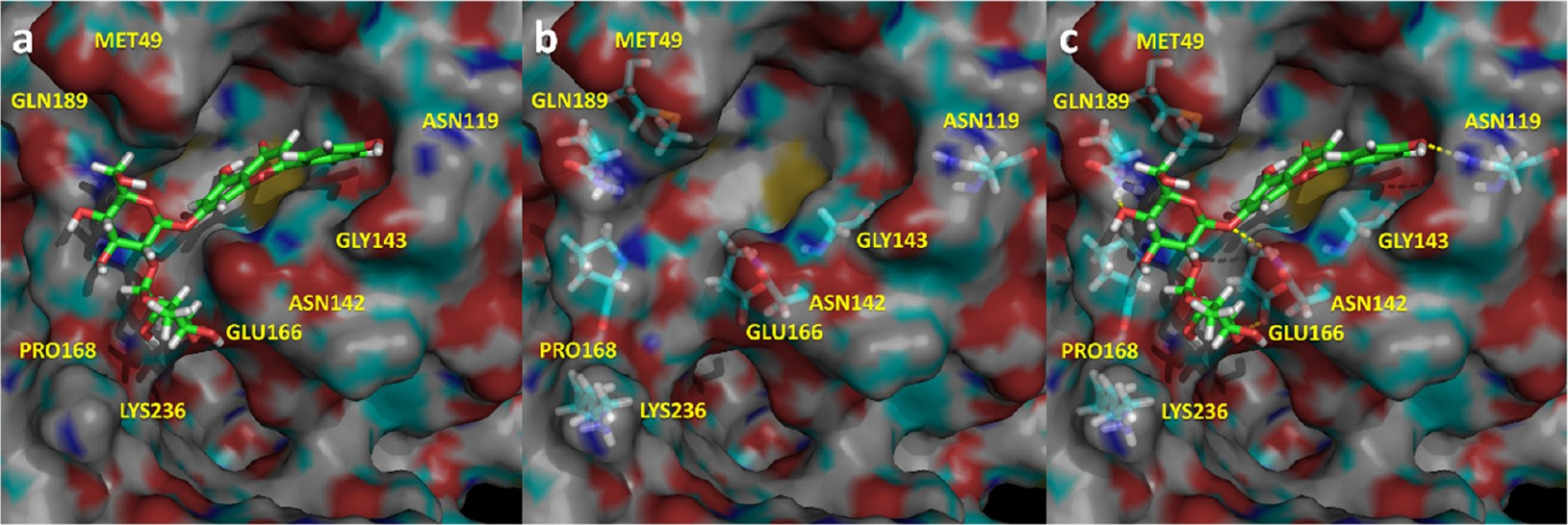
Spatial representation of the complex M^pro^-Rhiofolin in its last snapshot of the 1.5 μs molecular dynamics. (a) Ligand bound to the binding pocket. (b) Spatial distribution of the most important residues that interact with the ligand. (c) Ligand–protease hydrogen bonds in yellow.

The most active compound, (−) epigallocatechin gallate, (Figure 5) occupies subsites S1′, S1, and S2 establishing multiple interactions with different residues of the enzyme. Specifically, the ligand exhibits hydrogen bonds with Asp^48^, Cys^145^, His^164^, Glu^166^, and Asp^187^ together with a quadrupole–quadrupole interaction with His^41^, exhibiting complementary stereochemical features with the protease binding site.

Amentoflavone (Figure 6) occupies subsites S1′, S1, S2, and S4 establishing multiple hydrogen bonds with different residues of the enzyme including Cys^44^, Asn^142^, Cys^165^, and Glu^166^ together with a quadrupole–quadrupole interaction with His^41^.

Vitexin-2-*O*-rhamnoside (Figure 7) occupies subsites S1′ and S1, establishing multiple hydrogen bonds with different residues of the enzyme including Ser^46^, Ser^144^, Cys^145^, His^164^, Glu^166^, and Asp^187^ together with a quadrupole–quadrupole interaction with His^41^.

Aloin (Figure 8) also occupies subsites S1′ and S1, establishing multiple hydrogen bonds with different residues of the enzyme including Ser^46^, Met^49^, Ser^144^, Cys^145^, Met^165^, Asp^187^, and Gln^189^ together with a quadrupole–quadrupole interaction with His^41^.

Rhiofolin (Figure 9) occupies subsites S1′ and S2, establishing multiple hydrogen bonds with different residues of the enzyme including Met^49^, Asn^142^, Gly^143^, Cys^145^, Glu^166^, Gln^189^, and Lys^236^ together with a quadrupole–quadrupole interaction with His^41^.

**Figure 10:**
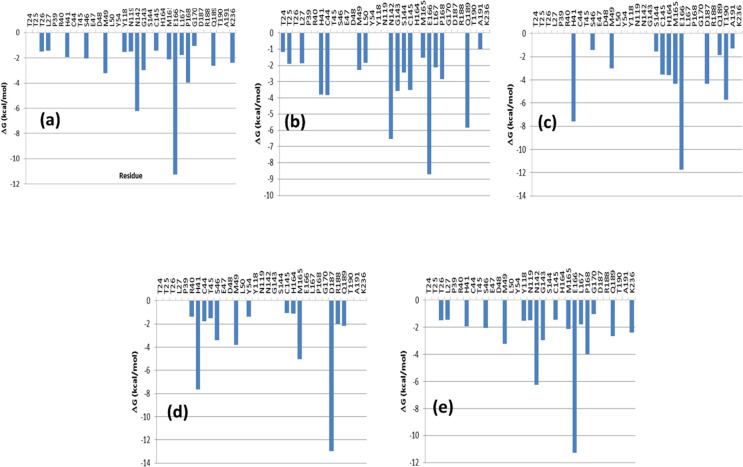
Residue decomposition of the binding free energy interaction for the diverse ligand-M^pro^ protease complexes found active. (a) (−) epigallocatechin gallate; (b) amentoflavone; (c) vitexin-2-*O*-rhamnoside; (d) aloin; and (e) rhoifolin.

This qualitative description of ligand–enzyme interactions can be further reinforced through the analysis of the individual residue contributions to the binding free energy shown in Figures 10a–e. Binding interactions for each residue–residue pair include three terms: van der Waals contribution, electrostatic contribution, and solvation contribution. The polar contribution of Δ*G*_solv_ was computed as in the case of the Δ*G*_bind_ using the GB model based on the parameters developed by Onufriev et al.^53^ All energy components were calculated using 25,000 snapshots corresponding to the last 100 ns of the full-length molecular dynamics run.

Analysis of Figures 10a–e shows that not all the compounds exhibit the same pattern of interactions, although there are specific residues relevant for binding that are common to all the compounds. Thus, these plots corroborate the involvement of dyad residues Cys^145^ and His^41^ in all the complexes. Moreover, the relevance of residue Glu^166^ and in some cases Asn^142^ or Asp^187^ as ligand-anchoring points is also underlined, as previously described. Actually, Glu^166^ is an important contributor to the binding energy of compounds like (−) epigallocatechin gallate, amentoflavone, vitexin-2-*O*-rhaMnoside, and rhoifolin, whereas the Asn^142^ is important for (−) epigallocatechin gallate, amentoflavone, and rhiofolin, whereas Asp^187^ is an important contributor for vitexin-2-O-rhamnoside and aloin. Interestingly, there are residues like Met^49^ or Pro^168^ that make a remarkable contribution to the binding energy of the ligands through van der Waals interactions.

Inspection of Table S1 of the Supporting Information also suggests the capacity of these ligands to form hydrogen bonds as a consequence of the high number of alcohol moieties they exhibit. Moreover, these molecules belong to the chemical class of polyphenols, considered to have antiviral, antibacterial, antioxidant, and anti-inflammatory activities. Specifically, diverse studies have investigated their potential antiviral efficiency against SARS-CoV-2 with varied results. Specifically, (−) epigallocatechin gallate^58−60^ and rhoifolin^25^ were previously identified as M^pro^ inhibitors, although other polyphenols may act as ligands of different enzymes.^63^

## 4 Conclusions

The need for the availability of compounds that can be used as therapeutic agents for the treatment of COVID-19 prompted to screen for approved drugs and natural products. Virtual screening is a cost-effective technique to screen for large libraries of compounds. The purpose of this work was to carry out virtual screening of the Selleck library of Natural Compounds using the M^pro^ protease of SARS-CoV-2 as the target aimed at identifying prospective antivirals. For this purpose, we carried out an ensemble docking of ca. 2000 compounds using seven different structures characterizing the plasticity of the M^pro^ binding pocket. Compounds showing binding to 6 or 7 of the diverse M^pro^ structures and with a scoring function above a threshold were selected for further analysis. After this process, we analyzed about 68 compounds that were screened according to the behavior of the binding free energy along a molecular dynamics process. Finally, 11 compounds were purchased and tested in vitro for their capability to inhibit the M^pro^ protease. The results show that 5 out of the 11 are active that gives a 45% success rate.

The resulting active five compounds were analyzed to identify residues responsible for their activity. Two analyses were done. On the one hand, one more qualitative from the inspection of the prospective bound conformation of the ligands inside the M^pro^ binding pocket and another more quantitative, where the binding free energy is decomposed in residue contributions. The results show that dyad residues Cys^145^ and His^41^ are involved in all the complexes and that Glu^166^ and Asn^187^ play an important role in the affinity of this group of inhibitors. Finally, other residues including Met^49^, Asn^142^, or Pro^168^, despite not being in direct contact with the ligands, interact with other residues playing a relevant role in defining the M^pro^ binding pocket.

## Supporting Information Available

Diverse information regarding the computational procedure followed, as well as details on the results produced in the in vitro studies (PDF)

## Notes

Coordinates of the seven structures of M^pro^ protease identified in the cluster analysis, as well as of the 11 ligand-M^pro^ complexes studied in this work are provided in the pdb format upon request to the authors.
