# Supplementary material for: Discovery of Diverse Natural Products as Inhibitors of SARS-CoV-2 Mpro Protease through Virtual Screening
Source: J Chem Inf Model. 2021 Nov 22;61(12):6094–106. doi: 10.1021/acs.jcim.1c00951 (PMC9931176; doi:10.1021/acs.jcim.1c00951)
Supplement: Supplementary file 1 — ci1c00951_si_001.pdf [file ci1c00951_si_001.pdf]

Supporting information

Discovery of Diverse Natural Products as inhibitors of SARS-CoV-2 M^{pro} Protease through Virtual Screening

Jaime Rubio-Martínez[1,*], Ana Jiménez-Alesanco[2,3], Laura Ceballos-Laita[2,4], David Ortega-Alarcón[2,3], Sonia Vega[2], Cristina Calvo[5,6], Cristina Benítez[5,6], Olga Abian[2,3,4,5,7], Adrián Velázquez-Campoy[2,3,4,5,8], Timothy Thomson[5,6,9], José Manuel Granadino-Roldán[10], Patricia Gómez-Gutiérrez[11], Juan J. Pérez[11].

[1]Department of Materials Science and Physical Chemistry, University of Barcelona and the Institut de Recerca en Quimica Teorica i Computacional (IQTCUB), 08028 Barcelona, Spain
[2]Institute for Biocomputation and Physics of Complex Systems (BIFI), Joint Units IQFR-CSIC-BIFI, and GBsC-CSIC-BIFI, Universidad de Zaragoza, 50018 Zaragoza, Spain
[3]Departamento de Bioquímica y Biología Molecular y Celular, Universidad de Zaragoza, 50009 Zaragoza, Spain
[4]Instituto de Investigación Sanitaria de Aragón (IIS Aragon), 50009 Zaragoza, Spain
[5]Centro de Investigación Biomédica en Red en el Área Temática de Enfermedades Hepáticas Digestivas (CIBERehd), 28029 Madrid, Spain
[7]Instituto Aragonés de Ciencias de la Salud (IACS), 50009 Zaragoza, Spain.
[6]Institute of Molecular Biology of Barcelona (IBMB-CSIC), 08028 Barcelona, Spain
[8]Fundación ARAID, Gobierno de Aragón, 50018 Zaragoza, Spain
[9]Universidad Peruana Cayetano Heredia, San Martín de Porres 15102, Perú.
[10]Departamento de Química Física y Analítica, Facultad de Ciencias Experimentales, Universidad de Jaén, Campus "Las Lagunillas" s/n, 23071, Jaén, Spain
[11]Department of Chemical Engineering. Universitat Politecnica de Catalunya- Barcelona Tech. Av. Diagonal, 647. 08028 Barcelona, Spain

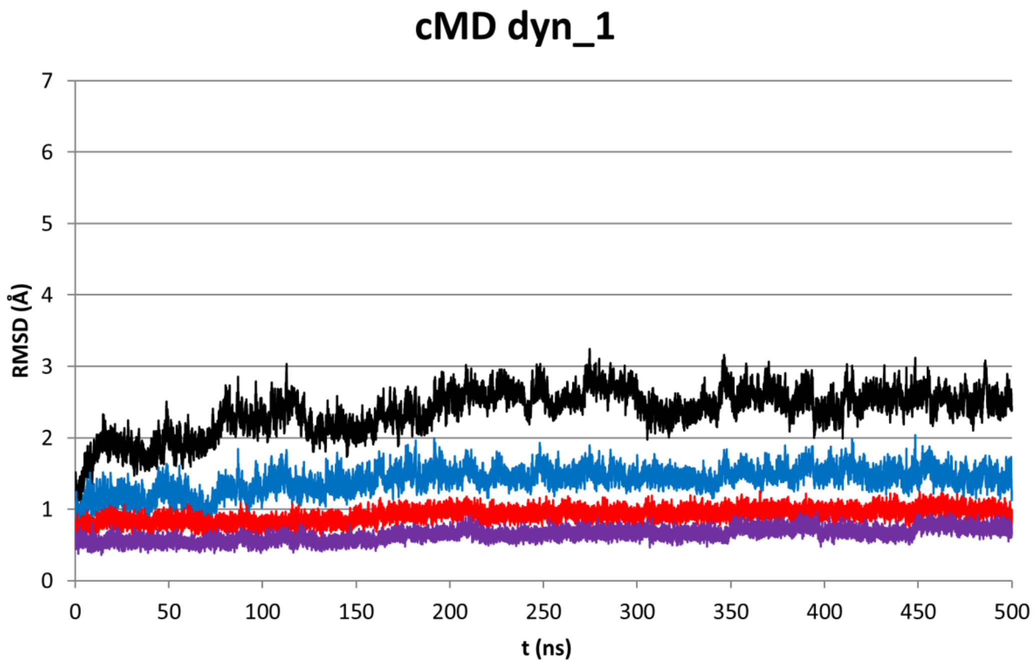
cMD dyn_1
RMSD (Å)
7
6
5
4
3
2
1
0
0
50
100
150
200
250
300
350
400
450
500
t (ns)

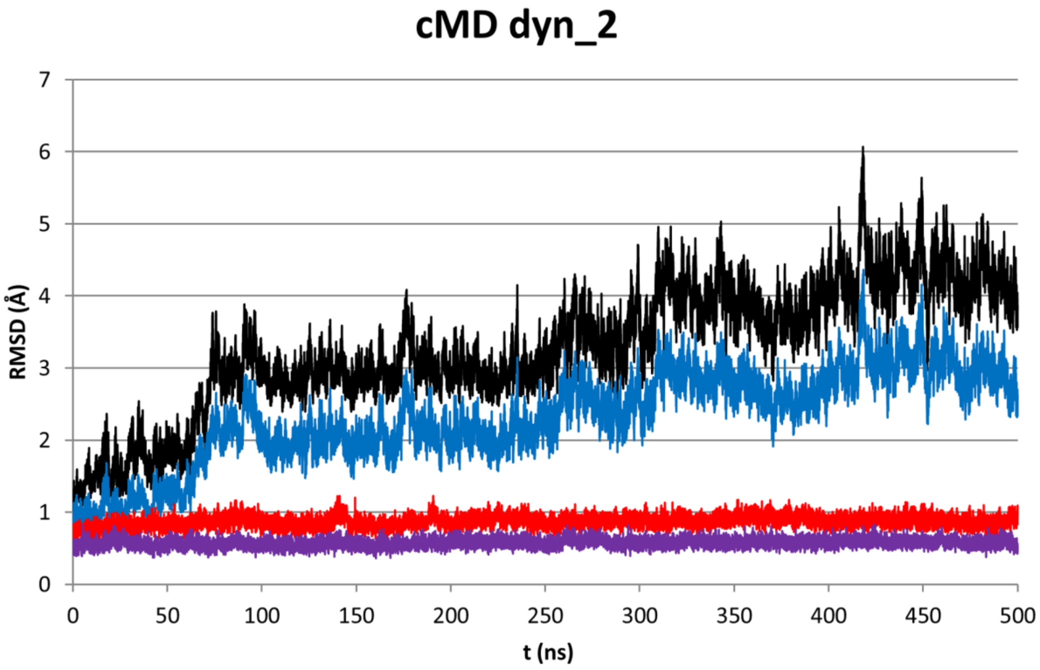
cMD dyn_2
RMSD (Å)
7
6
5
4
3
2
1
0
0
50
100
150
200
250
300
350
400
450
500
t (ns)

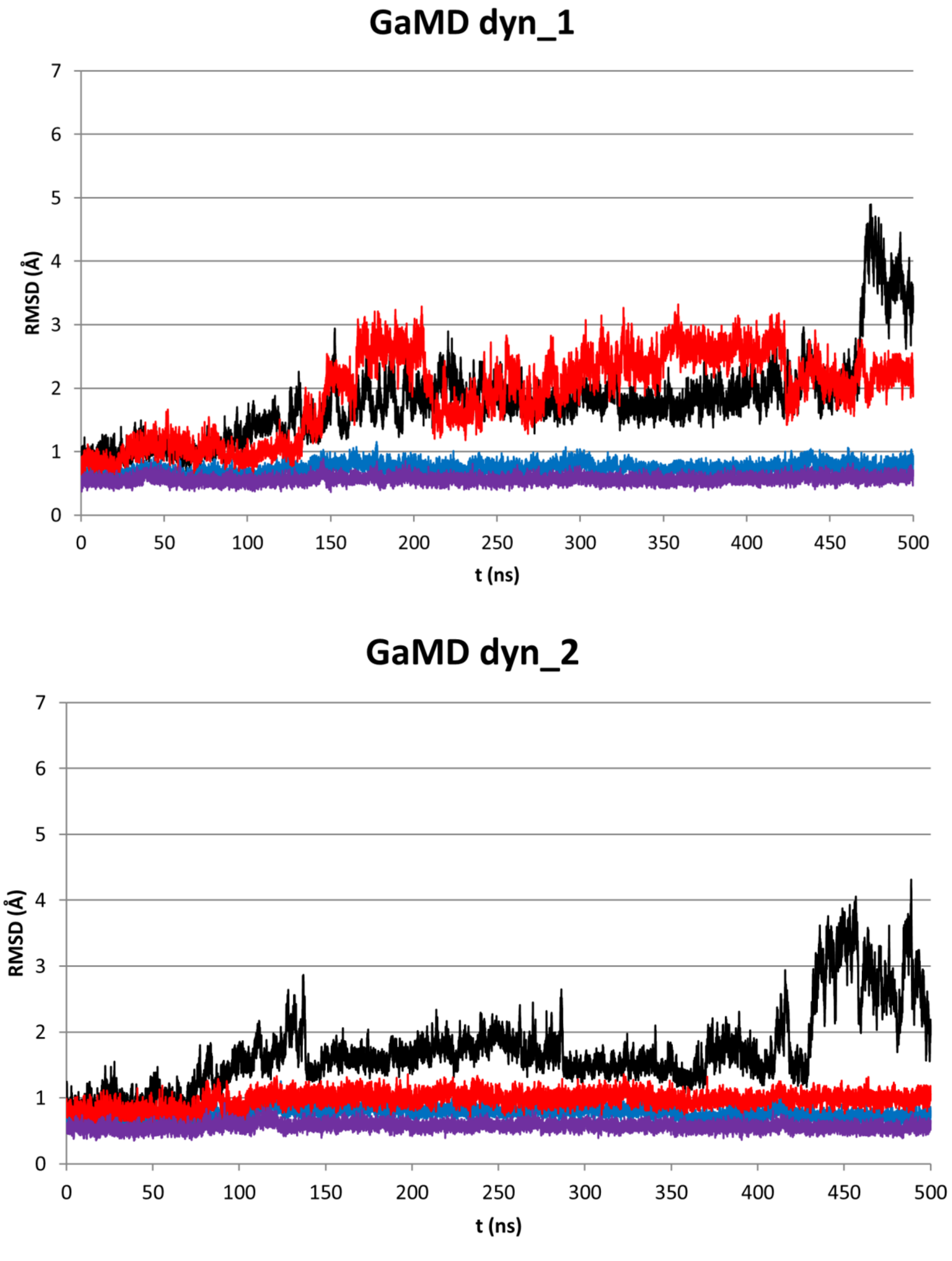

Figure S1. RMSD time evolution for each of the four steps (in different colors) used to determine protein residues with smallest RMSF. Black represents the first step, red represents the second step, blue represents the third and magenta fourth steps respectively.

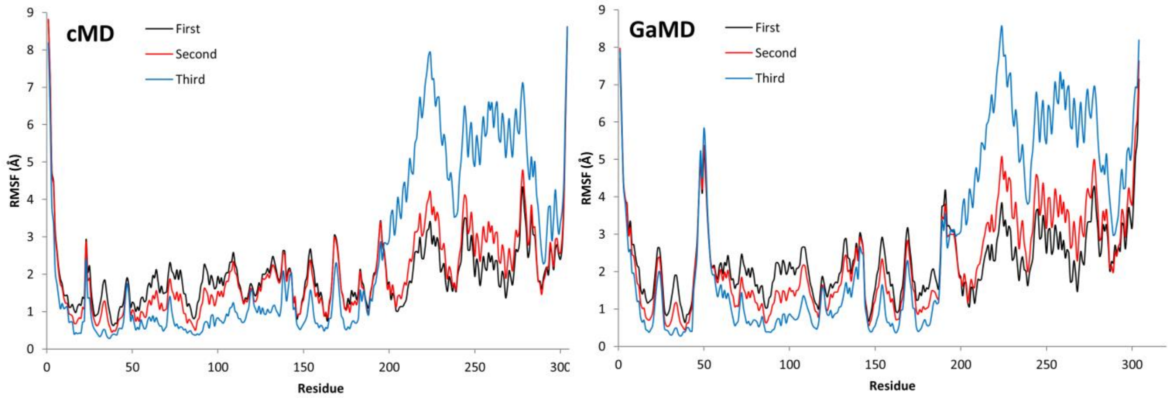

Figure S2. Variation of the RMSF with time for each of the steps used to determine protein residues with smallest RMSF.

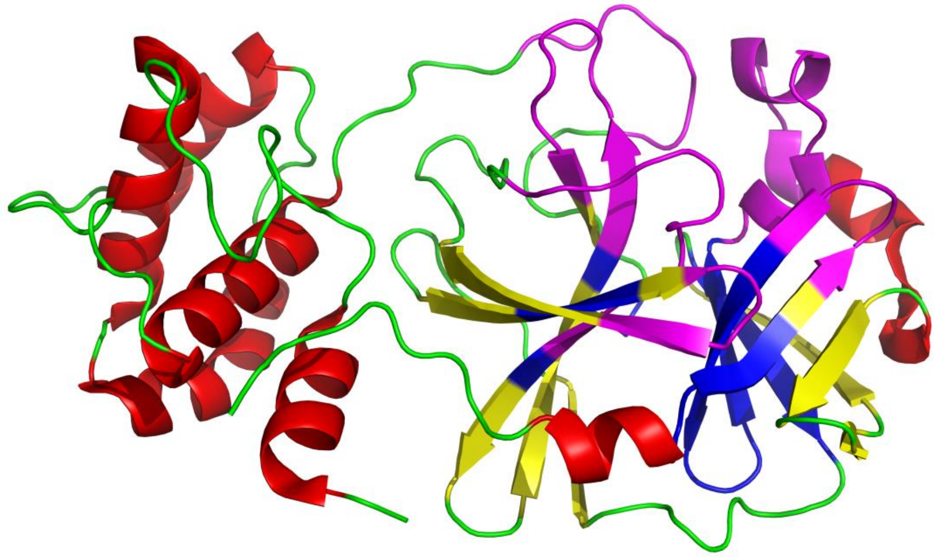

Figure S3. SARS-CoV-2 M^{pro} protease. Residues used to superpose the structures (low mobility) are depicted in blue. Residues used to do the clustering process and PC analysis (big mobility) are depicted in magenta.

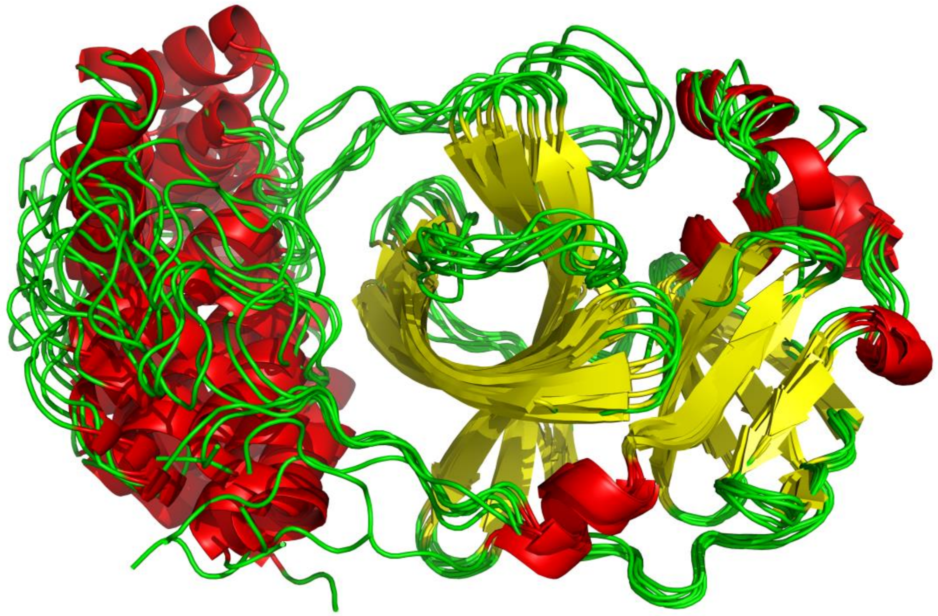

Figure S4. SARS-CoV-2 M^{pro} protease. Superposition of all seven representative structures using the binding site C_α with lowest RMSF as reference.

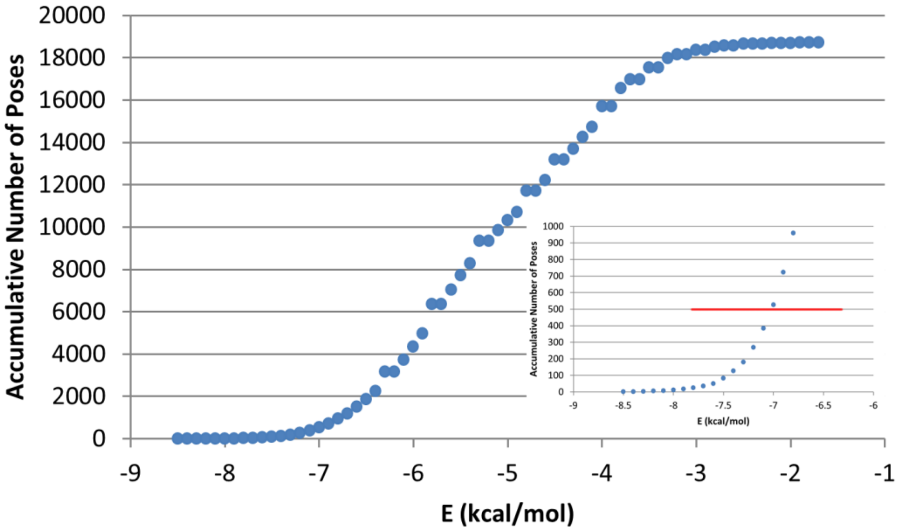

Figure S5. Plot of the accumulative number of structures produced in the docking process versus their binding energy for the most populated cluster identified in the cMD.

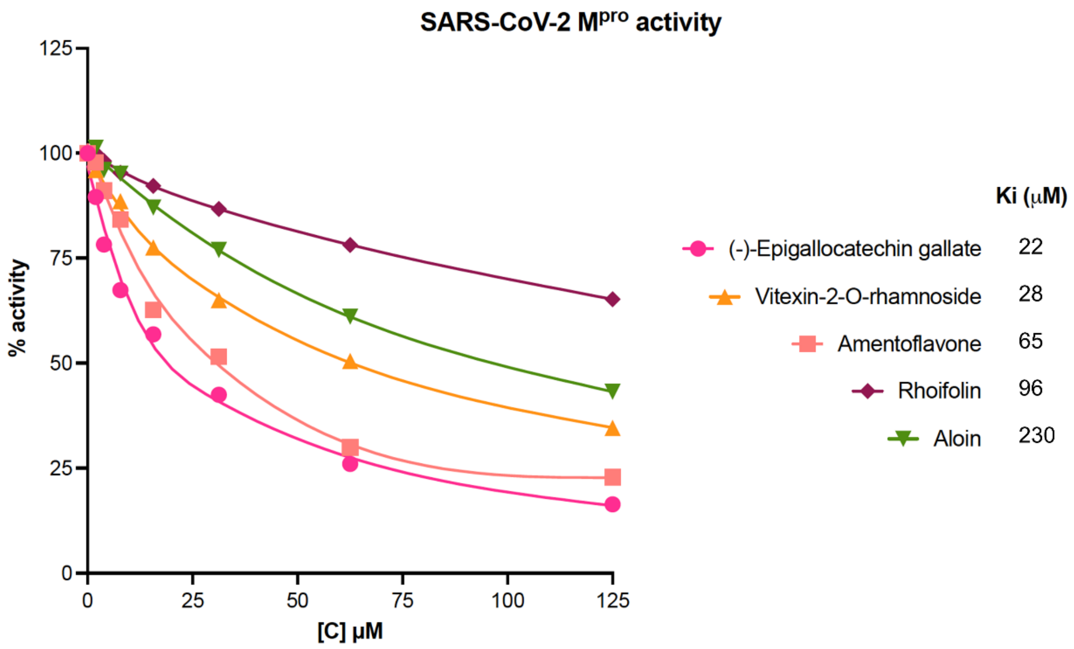

Figure S6. *In vitro* M^{pro} inhibitory activity of candidate compounds. Serial dilutions of the indicated compounds were used in a FRET-based activity assay with recombinant SARS-CoV-2 M^{pro} and a two-fluorophore substrate amenable to FRET. The substrate concentration-independent inhibition constant for each compound, K_i, was calculated as described in Materials and Methods. The substrate concentration-dependent IC_{50} was calculated by applying a least squares fit to a standard [inhibitor] vs. normalized response curve.

Table S1. Description of the most important hydrogen bonds obtained from the last 100 ns of the 1.5 μs MD trajectory.

System	Acceptor	Donor	Occupancy (%)
(-)Epigallocatechin galate	ASP187_OD2	Ligand_O10-H17	100.0
	ASP187_O	Ligand_O1-H8	64.8
	ASP187_O	Ligand_O9-H16	7.6
	ASP187_OD2	Ligand_O9-H16	47.6
	GLU166_O	ligand_O4-H11	78.4
	HIE164_ND1	ligand_O8-H15	61.2
	ASP48_OD2	ligand_O3-H10	51.2
	ASP48_OD1	ligand_O3-H10	47.2
	ASP48_OD2	ligand_O2-H9	48.6
	ASP48_OD1	ligand_O2-H9	46.0
Amentoflavone	CYS44_O	ligand_O6-H14	100.0
	GLU166_OE1	ligand_O8-H16	87.7
	GLU166_OE2	ligand_O8-H16	8.5
	ligand_O5	CYS145_N-H	63.3
	ASN142_OD1	ligand_O9-H17	32.6
	ligand_O9	ASN142_ND2-HD22	9.7
Vitexin-2-O-rhaMnoside	ASP187_O	ligand_O11-H27	44.8
	GLU166_OE1	ligand_O6-H14	41.4
	GLU166_OE2	ligand_O6-H14	40.9
	GLU166_OE2	ligand_O7-H15	37.9
	GLU166_OE1	ligand_O7-H15	37.6
	Ligand_O12	THR190_OG1-HG1	40.6
	Ligand_O13	THR190_OG1-HG1	10.8
	Ligand_O8	CYS145_N-H	20.6
Aloin	ASP187_O	ligand_O7-H14	95.2
	ASP187_O	ligand_O8-H15	85.9
	ASP187_OD2	ligand_O-H3	47.1
	ASP187_OD1	ligand_O-H3	32.4
	ligand_O7	GLN189_NE2-HE21	16.2
	ligand_O5	SER46_OG-HG	10.8
Rhoifolin	GLU166_OE2	ligand_O12-H28	96.0
	GLU166_OE2	ligand_O11-H27	16.1
	ligand_O1	GLN189_NE2-HE22	47.0
	ligand_O3	ASN142_ND2-HD22	24.2
	PRO168_O	ligand_O13-H29	22.8
	ASN119_OD1	ligand_O6-H14	13.8

All the compounds are >95% pure by HPLC analysis. The chromatograms of the active compounds are shown below: (a) (-) Epigallocatechin gallate; (b) Amentoflavone; (c) Vitexin-2-O-rhaMnoside; (d) Aloin; (e) Rhoifolin.

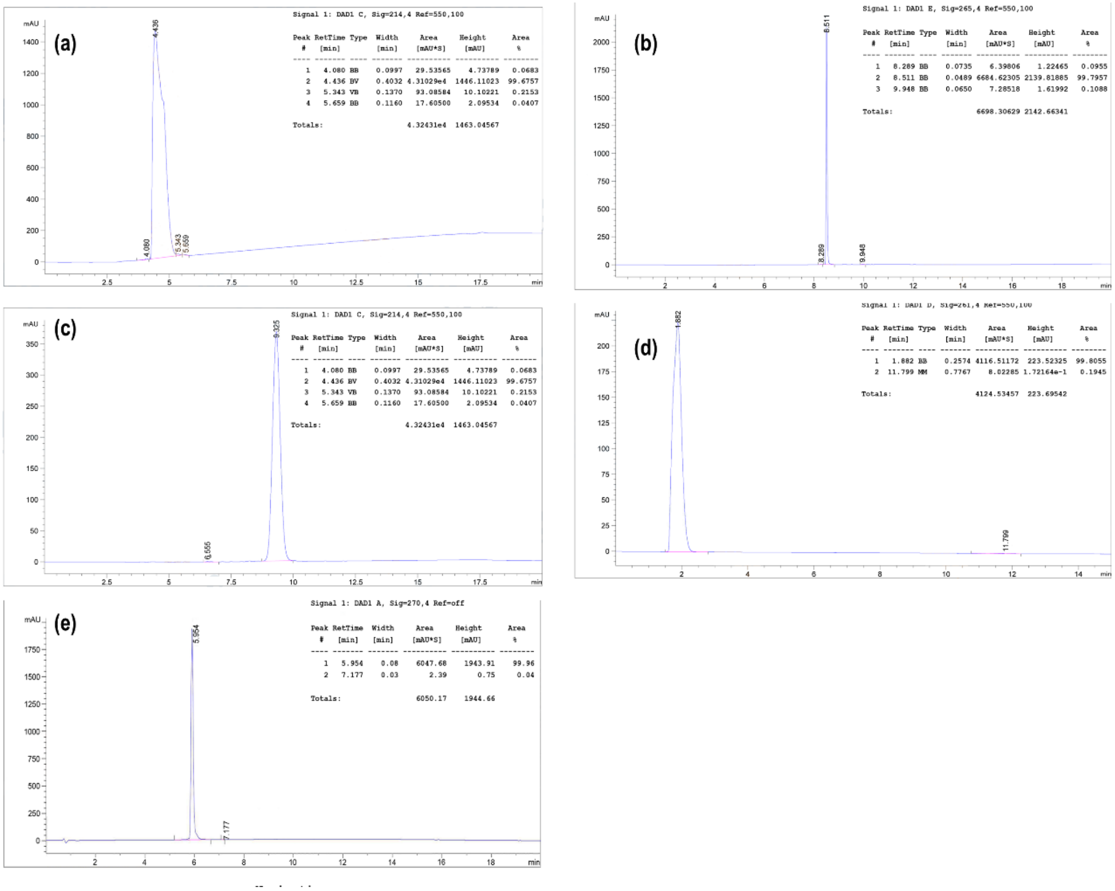

The asymmetric HPLC peak with a right shoulder observed in the epigallocatechin gallate chromatograph was further analyzed by means of mass spectrometry fragmentography experiments. As shown below epigallocatechin gallate is the predominant species (>99.2%) on HPLC. The sharp band, migrating with a peak at 4.4 min, yields epigallocatechin gallate characteristic fragments upon ionization. Epicatechin gallate is a minor species (<0.8%) on HPLC. The band, migrating with a peak at 5.34 min, yields epicatechin gallate characteristic fragments upon ionization. No other species or impurities are detected. We interpret that the shoulder on the original HPLC might have been a consequence of different running conditions that might have prevented a clear separation between epigallocatechin gallate and epicatechin gallate.

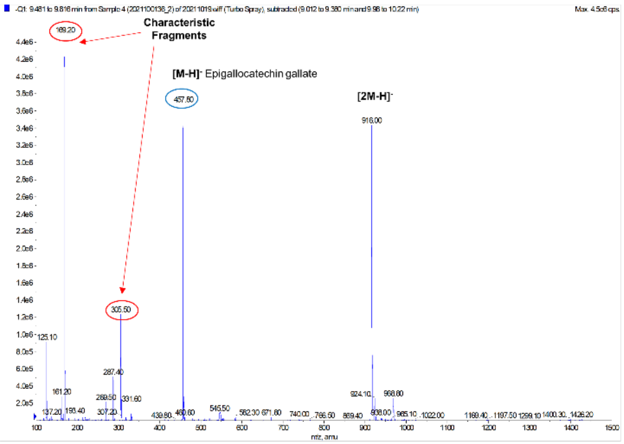

Epigallocatechin gallate: MS peak 4.4 min

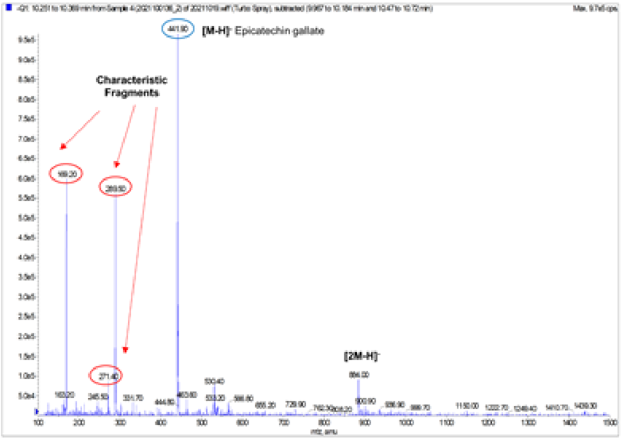

Epigallocatechin gallate: MS peak 5.34 min
